# Medial femoral condyle free flap for reconstructions of medication related osteonecrosis of the jaw

**DOI:** 10.1080/23320885.2026.2619311

**Published:** 2026-01-27

**Authors:** Derek C. Wenger, Caleb W. Brown, Phillip L. Nichols, Hannah Tan, Jeremy M. Powers

**Affiliations:** ^a^ Quillen College of Medicine, East Tennessee State University, Johnson City, TN, USA; ^b^ Division of Plastic and Reconstructive Surgery, Department of Surgery, Quillen College of Medicine, East Tennessee State University, Johnson City, TN, USA

**Keywords:** Free tissue transfer, mandibular reconstruction, medication-related osteonecrosis of the jaw, medial femoral condyle flap, vascularized bone flap

## Abstract

Medication-related osteonecrosis of the jaw (MRONJ) is a rare complication of antiresorptive therapy that may require free tissue reconstruction. A 57-year-old woman with metastatic breast cancer developed refractory mandibular MRONJ after intravenous zoledronic acid and underwent reconstruction with a medial femoral condylar (MFC) free flap. She recovered without complications and maintained normal function at two-year follow-up. This case supports the MFC free flap as a viable option for small, partial-thickness mandibular defects in selected patients with MRONJ.

## Introduction

Osteonecrosis of the jaw is a serious condition that results in exposure of necrotic mandible through eroded mucosal tissue. Patients often present with pain, swelling, exudation, or fistula formation [1]. A variety of etiologies have been identified, including adverse medication and radiation therapy effects, trauma, and infections such as syphilis, tuberculosis, and actinomycosis [2]. The leading cause of osteonecrosis of the jaw is medication-related adverse effects [3]. Medications frequently implicated in medication-related osteonecrosis of the jaw (MRONJ) include bisphosphonates (pamidronate and zoledronic acid), biologic antiresorptive medications (denosumab), and possibly antiangiogenic agents (bevacizumab, sunitinib) [2]. The risk of osteonecrosis is higher in patients treated with intravenous formulations as part of a cancer regimen [3,4]. The rates of MRONJ in patients with concurrent malignancy range from 1 to 8% compared to 0.01–0.3% in patients with osteoporosis [5,6]. The risk of MRONJ has also been observed to be dose- and duration-dependent with antiresorptive therapy. Poor dentition and invasive dental procedures further increase the risk of a patient experiencing MRONJ while on antiresorptive therapy [2,3]. The pathophysiology of bisphosphonate-induced osteonecrosis, while not completely understood, is thought to result from the deleterious effects of the medication on fibroblasts, keratinocytes, and immune function, leading to epithelial erosion and subsequent exposure of bone in the oral cavity, thus enabling bacterial invasion [2].

Correct staging of osteonecrosis is vital in disease treatment. A formal staging system for MRONJ was updated in 2022 by the American Association of Oral and Maxillofacial Surgery (AAOMS). Stage 0 includes patients lacking visible bone exposure but presenting with nonspecific symptoms such as jaw pain, altered sensation, or unexplained tooth mobility, along with radiographic abnormalities including alveolar bone loss, osteosclerosis, or thickening of the lamina dura. This stage is useful in determining whether a patient is at high risk for developing more severe disease, even if they have not yet met the criteria for advanced MRONJ. Stage 1 is characterized by exposed or probeable bone in the absence of pain or infection. In stage 2, patients exhibit exposed bone with clinical signs of infection, such as pain, swelling, or purulence. Stage 3 involves exposed bone and infection accompanied by one or more serious complications, including pathologic fracture, extraoral fistula, or necrosis extending beyond the alveolar bone into adjacent structures such as the sinus or inferior border of the mandible [7].

Due to the relative rarity of MRONJ, there is still discussion regarding the best treatment approach. Management strategies for osteonecrosis of the jaw (ONJ) outlined by AAOMS range from conservative to highly invasive interventions. Nonoperative options emphasize local wound care, antimicrobial rinses, systemic antibiotics, and removal of mobile sequestra, with the goals of stabilizing disease, relieving pain, and preventing infection [7]. In more advanced cases, adjunctive measures such as hyperbaric oxygen therapy or the PENTO regimen (pentoxifylline and tocopherol) have been utilized, though evidence of efficacy remains limited [8]. For patients who fail conservative therapy or present with progressive disease, AAOMS algorithms support operative approaches tailored to the disease stage, ranging from marginal resection above the mandibular canal in early disease to segmental resection with free flap reconstruction in more advanced cases [7].

Uncertainty remains regarding whether surgery should be pursued early *versus* after exhaustion of conservative measures, and decisions are best individualized through shared decision-making. Treatment regimens for MRONJ differ slightly from ORN. Although clinicians most often recommend conservative treatment first for both pathologies, surgical management is generally accepted earlier in the treatment process for MRONJ. This is partially due to the lack of robust evidence supporting the PENTO regimen that is often used in ORN. Free flap reconstruction is offered for patients whose disease is refractory to other options or who present initially with severe cases [9]. We present a case in which early free flap reconstruction was performed due to patient-specific pathology and preference, with a favorable long-term outcome.

## Patient

**Figure 1.**
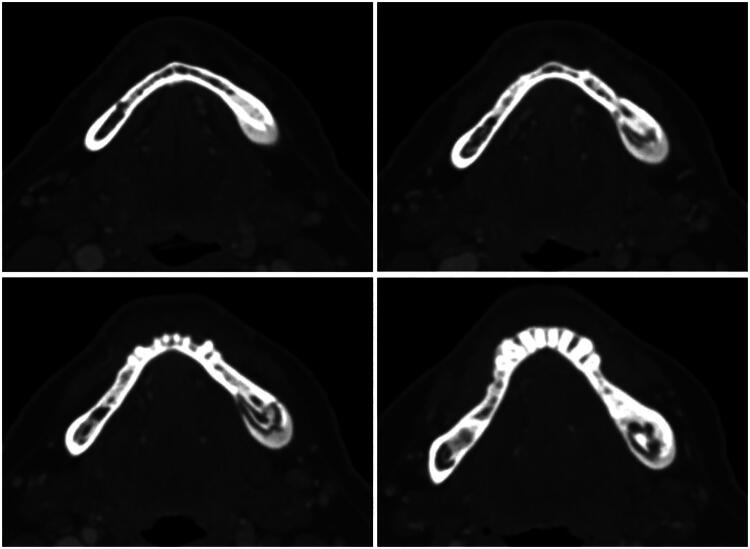
Computed tomography (CT) of the mandible demonstrating an osseous lesion involving the left paramedian body with features suspicious for chronic osteomyelitis. Note the partially extruded bony sequestrum and cortical irregularity, consistent with medication-related osteonecrosis of the jaw (MRONJ).

A 57-year-old female presented for evaluation of MRONJ after undergoing treatment with intravenous zoledronic acid every six weeks for one year as part of her regimen for metastatic breast cancer. She developed pain and swelling of her left posterior jaw and sought treatment from oral surgery. Her zoledronic acid was discontinued, and she was prescribed Augmentin and underwent multiple intra-oral debridements of necrotic bone. Computed tomography (CT) of the mandible demonstrated an osseous lesion involving the body of the left paramedian mandible, suspicious for chronic osteomyelitis with partially extruded bony sequestrum, suggestive of osteonecrosis (Figure 1). Due to symptom progression, she was referred to plastic surgery to discuss definitive interventions. After reviewing available options—including continued debridement, hyperbaric oxygen therapy, and marginal resection with free flap reconstruction—the patient requested surgical reconstruction as her symptoms were refractory to conservative management and she wanted to avoid the possibility of multiple procedures.

Multiple reconstructive options, including a vascularized medial femoral condyle (MFC) free flap and a free fibula flap, were discussed with the patient as part of preoperative counseling. Given the limited, partial-thickness nature of the mandibular defect and the desire to minimize donor-site morbidity, the MFC free flap was selected as the primary reconstructive option. The free fibula flap was considered an alternative should a greater volume of bone be required or if intraoperative findings necessitated a different reconstructive strategy.

**Figure 2.**
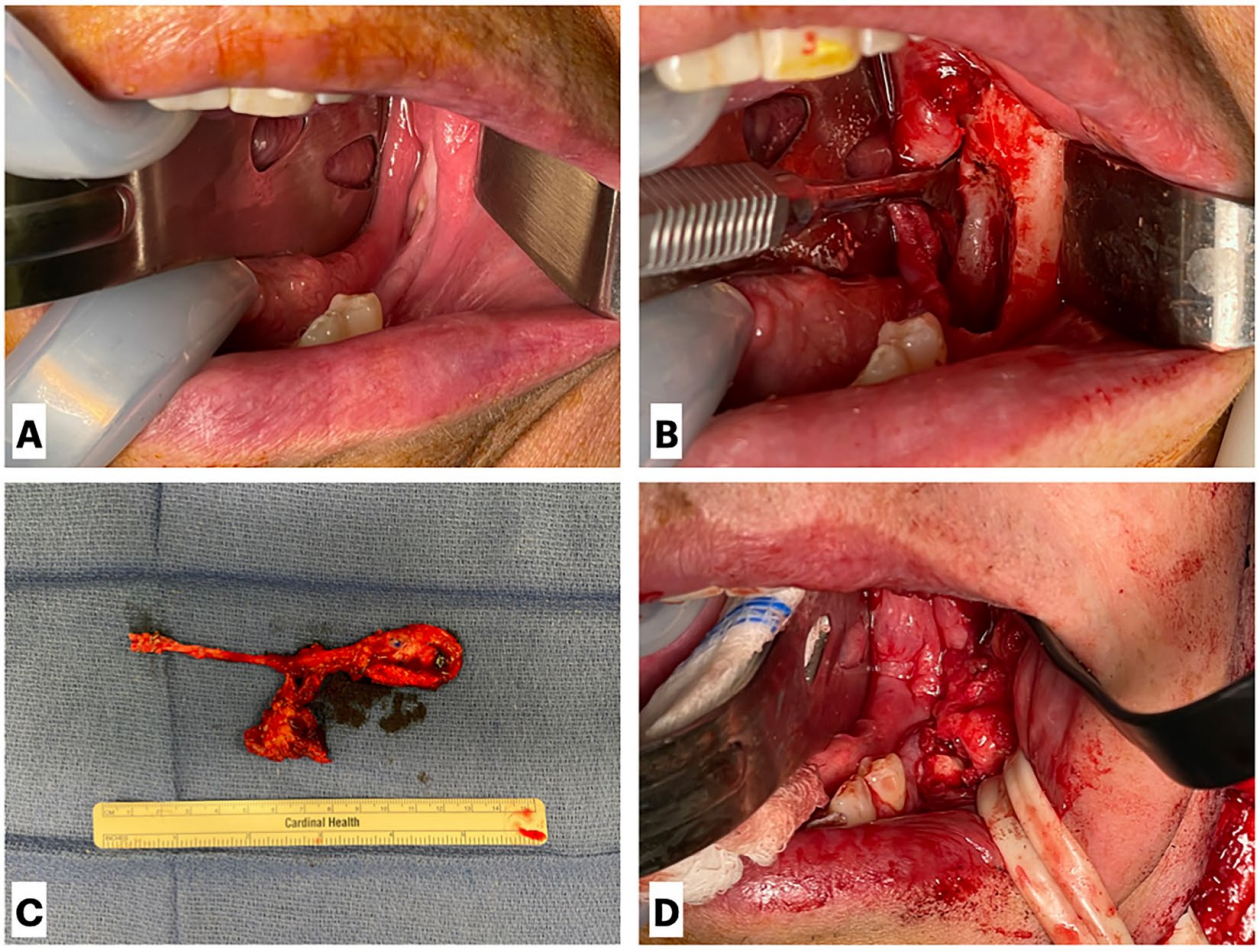
Intraoperative views of medial femoral condyle (MFC) free flap harvest and inset. (A) Visualization of necrotic mandibular defect prior to surgical debridement. (B) Intraoral defect following debridement to healthy, bleeding bone, measuring ∼2 cm in length and 1.5 cm in depth. (C) Harvest of a 2 × 2 cm cortical bone segment, a 3 × 4 cm periosteal segment, and a small cuff of vastus medialis supplied by branches of the descending geniculate artery. (D) Final inset of the vascularized MFC free flap into the mandibular defect.

Intraoperatively, the intraoral defect was debrided until healthy, bleeding bone was encountered, measuring ∼2 cm in length and 1.5 cm in depth. The facial artery and vein were dissected and prepared for use as recipient vessels, and a tunnel was created to the intraoral defect. Attention was then directed to the right leg, where dissection was carried out to adequately expose the MFC free flap. The descending geniculate artery pedicle and its branches to the corticoperiosteal segment were identified and dissected back to the superficial femoral artery. A 2 × 2 cm segment of cortical bone, a 3 × 4 cm segment of periosteum, and a small cuff of vastus medialis supplied by branches of the descending geniculate artery were isolated and harvested. The flap was shaped to fit the intraoral defect, and the pedicle was tunneled into position. The cortical bone was secured within the bony defect using 7-mm KLS self-drilling screws. To provide robust vascularity and thicker coverage, the vastus medialis was draped over the corticoperiosteal segment, and the oral mucosa was closed primarily over the flap. Microvascular anastomoses were then performed, connecting the descending geniculate artery and vein to the facial artery and vein (Figure 2). Finally, the leg and neck incisions were closed with drains in place with care taken to avoid compression or overlap of the vascular pedicle in the neck, and an implantable Doppler probe was left for postoperative monitoring.

## Results

**Figure 3.**
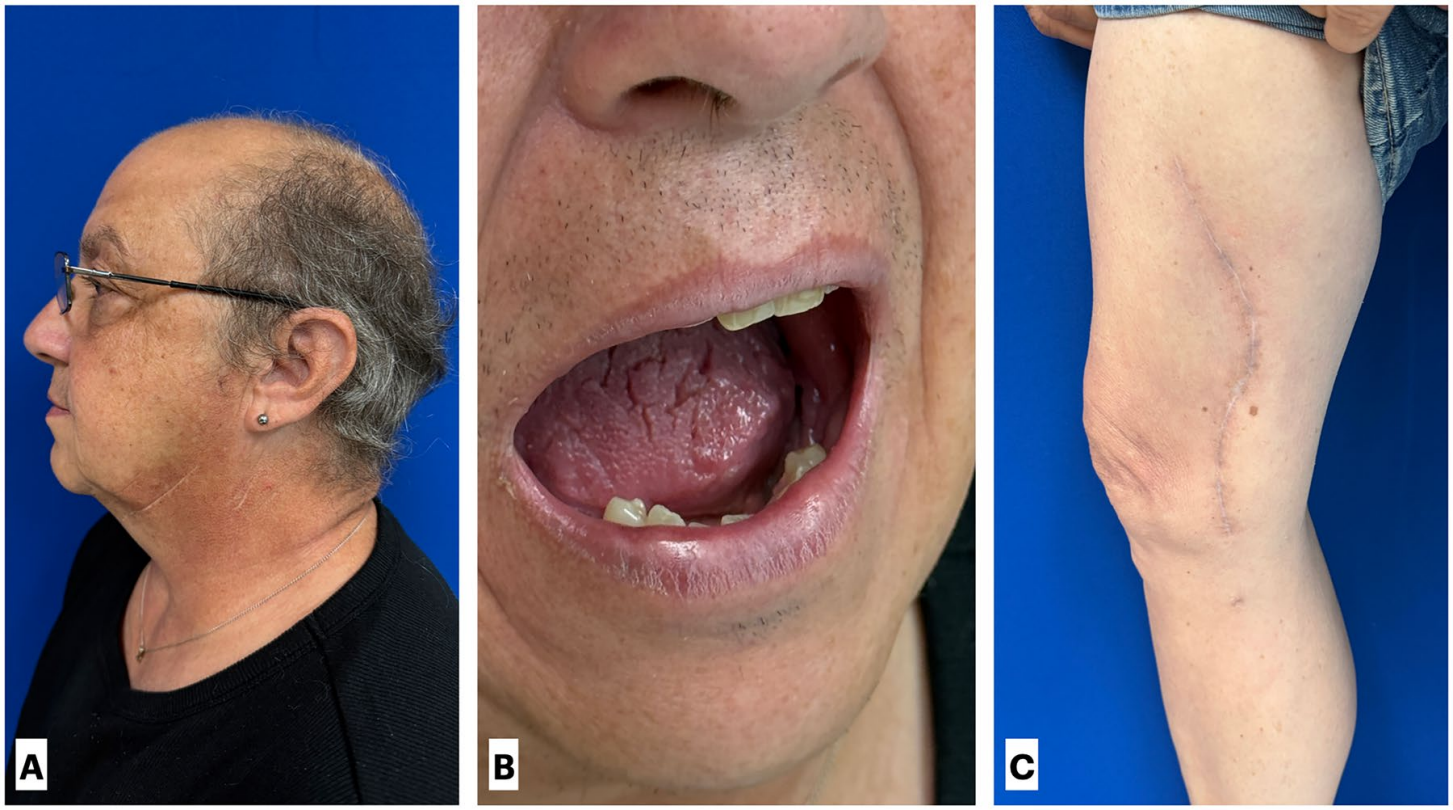
Two-year postoperative follow-up demonstrating durable reconstruction and functional recovery. (A) Profile view showing satisfactory contour and symmetry of the mandible. (B) Intraoral view demonstrating intact mucosal coverage without evidence of dehiscence or recurrent osteonecrosis. (C) Right medial thigh donor site with well-healed donor site scar.

The patient tolerated the procedure well without postoperative complications and was discharged home four days later and was permitted weight bearing as tolerated. Drains were maintained at hospital discharge and subsequently removed in the outpatient setting once output was <33 mL/day for two consecutive days. During outpatient follow-up, the patient progressed appropriately, with no evidence of flap failure, wound breakdown, or donor-site morbidity, and complete healing of the intraoral mucosal defect. Follow-up has been completed for two years, with normal oral function (Figure 3). At the most recent follow-up, the patient demonstrated no appreciable functional limitation or movement restriction of the donor knee.

## Discussion

With the advent and rapid advancement of microsurgical flap techniques and options over the past 50 years, the predominant reconstructive approach has shifted from the classical ‘ladder’ model, adapted from wound closure principles, to the more modern ‘reconstructive elevator’. Whereas the ladder implies a rigid, stepwise escalation from simple to complex techniques, the elevator emphasizes parallel consideration of all reconstructive options from the outset, selecting the approach that best balances durability, function, and aesthetics. This framework is particularly important in the head and neck, where functional and cosmetic outcomes are closely linked, and the simplest repair may not provide a reliable long-term result. By moving directly to the most definitive reconstructive option, the elevator avoids the delays, cost, and morbidity that can arise from prolonged conservative intervention [10].

The MFC free flap represents a newer, versatile option for smaller mandibular defects. It can be harvested to include cortical bone, cartilage, cancellous bone, adjacent muscle tissue, and periosteum. The option to harvest a pure periosteal or corticoperiosteal flap is especially useful in cases where bone regeneration is necessary while also offering robust coverage and vascularity. The inner cambium layer of the periosteum contains skeletal progenitor cells that promote cartilage and bone formation and support long-term ossification when revascularized. This osteogenic potential assists in achieving durable reconstruction of small segmental defects [11].

The versatility of the MFC free flap has been demonstrated across numerous reconstructive contexts beyond the mandible. In the upper extremity, it has been used for lunate osteonecrosis [12], capitate avascular necrosis [13], metacarpal and digital bone defects [14,15], traumatic thumb loss [16], and salvage of failed ulnar allografts [17], providing durable bone healing and restoration of function. In the lower extremity, applications include talar reconstruction with chondro-osseous and periosteal components [18] and calcaneal salvage after osteomyelitis [19], where chimeric designs facilitate joint preservation and weight-bearing capacity. In the head and neck, the flap has been reported for subtotal auricular reconstruction [20], frontal sinus repair [21], laryngotracheal reconstruction [22], alveolar arch repair [23], and mandibular osteoradionecrosis [24,25]. Collectively, these reports highlight the adaptability of the flap, with its ability to supply bone, periosteum, cartilage, and muscle tailored to the requirements of diverse defects. While prior studies have described its use in osteoradionecrosis of the jaw [24,25], to our knowledge, no reports have specifically documented its application in MRONJ.

When comparing the MFC free flap to other bony flaps used to reconstruct head and neck defects, emerging evidence suggests comparable outcomes to established workhorse flaps. A 2024 meta-analysis evaluating 582 MFC free flaps found a 2% flap failure rate (1% when analyzing head and neck–only defects) and a 4% donor-site morbidity rate [26]. The MFC free flap resulted in a similar rate of gait disturbance compared with the fibula flap [27], but a much lower overall donor-site morbidity rate, as fibula flaps have a reported rate of 30% [28]. The MFC free flap also carries a lower risk of vascular compromise to critical structures, as it harvests terminal branches of the descending geniculate artery, whereas the fibula relies on the peroneal artery, which supplies collateral flow to the foot [26,27,29]. Additionally, the MFC free flap eliminates the risk of great toe ‘clawing’ associated with detachment of the flexor hallucis longus muscle from the fibula. Another rare complication of free fibula flap harvest is foot drop due to common and/or deep peroneal nerve injury, which is not a consideration with MFC free flap harvest [28]. The MFC free flap donor site also often provides a better cosmetic result, as the incision is placed on the medial thigh rather than the lower leg. By contrast, the scapular osteocutaneous flap is associated with the lowest relative donor-site morbidity, typically limited to mild shoulder function changes that rarely affect daily activities, whereas the iliac crest flap offers good bone stock and a hidden scar but carries higher risks of chronic pain, contour deformity, sensory deficits, and hernia formation [30].

Common donor-site complications of the MFC free flap include paresthesia, knee pain, and decreased range of motion, which frequently resolve within weeks of surgery. Severe complications are rare, with femur fractures reported in 3 of 582 patients; all occurred in patients with prior bony trauma requiring fixation in the knee or in cases where larger bony segments extending into the femoral shaft were harvested. Other rare but serious complications include superficial femoral artery injury and medial collateral ligament (MCL) injury, each reported in one patient. Limitations of the flap include the smaller maximum harvest size of ∼7 cm of bone, a short vascular pedicle of 6–10 cm, and tenuous vessels supplying the skin paddle [26,28].

While MFC free flap has gained increasing use in head and neck reconstruction, comparative evidence demonstrating its superiority over other vascularized osseous free flaps is currently lacking. The free fibula flap remains the most utilized reconstructive option for MRONJ, with systematic reviews reporting high success rates and low recurrence following free flap reconstruction. In a systematic review, fibula-based reconstruction accounted for 80.76% of cases, with an overall free flap success rate of 96.16% [31]. In this context, the MFC free flap is best considered a complementary option within the reconstructive armamentarium rather than a replacement for established techniques. Its thin, well-vascularized corticocancellous bone and relatively low donor-site morbidity may offer advantages for select patients with small to moderate defects in compromised tissue beds; however, larger comparative studies are needed to better define its role relative to more commonly utilized free flaps. Furthermore, review of the recent literature suggests that the MFC free flap demonstrates comparable outcomes with respect to endosseous dental implant placement, although available data are limited to small cohorts [32–34].

In this case, the MFC free flap was a viable option due to both the clinical presentation and the patient’s treatment goals. The patient met criteria for stage 2 MRONJ based on the presence of exposed necrotic bone with evidence of infection according to AAOMS guidelines [7]. She remained symptomatic despite extensive conservative management, including antibiotic therapy, repeated debridement, and hyperbaric oxygen treatment, consistent with recalcitrant disease. Prior studies have demonstrated that mandibulectomy with free flap reconstruction is an effective treatment strategy for patients with stage 3 and refractory stage 2 bisphosphonate-related osteonecrosis of the jaw, supporting the decision for vascularized reconstruction in this case [35]. Additionally, considering unsuccessful prior therapies, the patient expressed a preference for definitive surgical reconstruction. The MFC free flap was selected because of the small, partial-thickness nature of the defect. The periosteal and vastus medialis components of the flap also provided the benefits of enhanced bone regenerative capacity and increased soft tissue coverage.

This report has several limitations inherent to its design. As a single-patient case report, the findings may not be generalizable to all patients with MRONJ or to other reconstructive indications. Functional outcomes were assessed clinically rather than through validated outcome measures. Additionally, late postoperative imaging was not available to radiographically confirm osseous integration of the flap, as further imaging was not clinically indicated following uncomplicated healing. Finally, this report does not provide comparative outcomes between the MFC free flap and other osseous free flaps. Larger, comparative studies are needed to better define functional outcomes and the relative role of the MFC free flap in mandibular reconstruction for MRONJ.

## Conclusion

Osteonecrosis of the jaw is a rare but debilitating adverse effect of antiresorptive and antiangiogenic medications, with less frequent causes including radiation, trauma, and infection. Treatment may be conservative, but surgical management is often necessary for complete resolution of symptoms. Ideal regimens incorporate infection prevention, pain control, and restoration of healthy bone. Discussion persists regarding the most appropriate initial approach to treating osteonecrosis of the jaw.

The MFC free flap is a newer and comparably successful free flap option for reconstructing head and neck defects. It offers versatility that other options lack and is associated with potentially lower rates of donor-site complications. Though rare, severe complications can occur, and careful planning and discussion with patients are critical before proceeding with this operation. Additional research comparing outcomes between the MFC free flap and other bony flaps is needed to establish clearer standards for when this flap is the most appropriate option for reconstruction.

## Data Availability

The data that support the findings of this case report are not publicly available due to privacy restrictions.
